# A taxonomic reassessment of the genus *Balsamia* from China

**DOI:** 10.3897/mycokeys.67.50068

**Published:** 2020-06-04

**Authors:** Yu-Yan Xu, Xiang-Yuan Yan, Ting Li, Li Fan

**Affiliations:** 1 College of Life Science, Capital Normal University, Xisanhuanbeilu 105, Haidian, Beijing 100048, China Capital Normal University Beijing China

**Keywords:** Ascomycota, Helvellaceae, Hypogeous fungi, phylogeny, taxonomy

## Abstract

Molecular analysis of the genus *Balsamia* was conducted with ITS and 28S sequences available, including newly gained sequences from Chinese specimens. Combined with the morphological examinations, a new hypogeous species, *Balsamia lishanensis* was described and illustrated from North China, which is morphologically characterized by reddish brown ascomata covered with fine warts, the whitish gleba with numerous small chambers, 3–5 layers peridium with reddish brown polygonal cells and the smooth and regular ellipsoid ascospores with one large oil drop. Two species previously described as *Barssia* were transferred to *Balsamia*. *Balsamia platyspora* was confirmed to be in existence in China based on newly collected specimen. A key to the Chinese *Balsamia* species was provided.

## Introduction

The genus *Balsamia* Vittad. (*Helvellaceae*, *Pezizales*), with *B. vulgaris* Vittad. as the type species, was established in the early 19^th^ century (Vittadini 1831), usually forming ectomycorrhizae with both broad leaf and conifer trees (Southworth et al. 2018; Hansen et al. 2019). Geographically, *Balsamia* species are widely distributed across Europe, North America, North Africa and Asia in the temperate regions of the northern hemisphere (Liu and Tao1990; Pegler et al.1993; Southworth et al. 2018; Hansen et al. 2019). Until now, nine *Balsamia* species have been reported from Europe (Vittadini 1831; Tulasne and Tulasne 1843; Berkeley 1844; Tulasne and Tulasne 1851; Schulzer 1870; Petitberghien 1966; Ławrynowicz and Skirgiełło 1984; Kaounas et al. 2015; Hansen et al. 2019), twelve from North America (Southworth et al. 2018), and one from North Africa (Crous et al. 2014; Hansen et al. 2019) In China, this genus is poorly understood as only one species *Balsamia platyspora* Berk. is reported, based on morphological evidence (Liu and Tao 1990).

Recently, two new species of the genus *Barssia* have been described from China (Xu et al. 2018), their taxonomic position, however, needs to be reassessed because Hansen et al. (2019) synonymized *Barssia* under *Balsamia* based on their phylogenetic analysis from three loci (*28S*, *RPB2*, *EF-1α*) and morphological studies. More recently, an un-described *Balsamia* species is recognized when we check the specimens newly collected from north China. In this paper, both the molecular analyses and morphological examinations are conducted for the Chinese samples,, and our aims are:1) to illustrate the position of Chinese *Balsamia* species based on ITS and 28S sequences newly obtained from Chinese *Balsamia* collections with distinct features in this study as well as recently published and used sequences ; 2) to give a detailed characterization of a new species based on morphological features and phylogenetic evidences.

## Materials and methods

### Morphological studies

Collections were obtained and photographed in the field from Shanxi regions in China, and they were dried and deposited in BJTC (Herbarium, Biology Department, Capital Normal University). One specimen was studied from HMAS (Herbarium Mycologicum Academiae Sinicae, Institute of Microbiology, Chinese Academy of Sciences). Macroscopic characters were recorded from fresh specimens. Microscopic characters were observed in thin sections of dry specimens mounted in 3% KOH, Melzer’s reagent (Dring 1971) or 0.1% (w/v) cotton blue in lactic acid. Thirty mature ascospores were measured, and the symbol Q is used to indicate length/width ratios of ascospores in side view.

### DNA extraction, PCR amplification and DNA sequencing

Herbarium specimens were crushed by shaking for 30 s at 30 Hz 2–4 times (Mixer Mill MM 301, Retsch, Haan, Germany) in a 1.5 ml tube together with one 3 mm diam. tungsten carbide ball, and total genomic DNA was extracted using the modified CTAB method (Gardes and Bruns 1993). The internal transcribed spacer (ITS) region of nuclear ribosomal DNA (nrDNA) was amplified using primers ITS1f/ITS4 (White et al. 1990; Gardes and Bruns 1993). The 28S large subunit nrDNA (nrLSU) region was amplified using primers LR0R/LR5 (Vilgalys and Hester 1990). PCRs were performed in a volume of 50 μl consisted of 4 μl of DNA template; 2 μl of (10 μM) per primer; 25 μl 2× Master Mix (Tiangen Biotech Co., Beijing). The procedure for PCR reaction was: an initial denaturation at 94 °C for 3 min; followed by 35 cycles at 94 °C for 30 s, 55 °C for 45 s, 72 °C for 1 min; and a final extension at 72 °C for 10 min. The PCR products were sent to Beijing Zhongkexilin Biotechnology Co. Ltd. (Beijing, China) for purifying, sequencing and editing. Validated sequences are stored in the NCBI database (http://www.ncbi.nlm.nih.gov/) under the accession numbers provided (Table 1). The other sequences used in the molecular phylogenetic analysis were downloaded from the NCBI database (Suppl. material 1).

**Table 1.** Information on newly generated DNA sequences used in this study.

**Fungal taxon**	**Specimen voucher**	**Locality**	**ITS**	**28S**
*Balsamia lishanensis*	BJTC FAN587	Shanxi, China	MT232721	MT232903
	BJTC FAN591	Shanxi, China	MT232899	MT232911
	BJTC FAN676	Shanxi, China	MT232907	MT232902
	BJTC FAN689	Shanxi, China	MT232905	MT232914
	BJTC FAN697	Shanxi, China	MT232908	MT232912
	BJTC FAN714	Shanxi, China	MT232901	MT232913
	BJTC FAN1010	Shanxi, China	MT232900	MT232910
	HMAS 97115	Gansu, China	MT232904	MT232909
*Balsamia platyspora*	BJTC FAN557	Shanxi, China	MT232906	MT229143

### Phylogenetic analyses

Two datasets, ITS and 28S, were compiled to identify *Balsamia* species and investigate relationships among species. The taxa *Tuber anniae* and *T. bellisporum* were selected as outgroups. The ITS and 28S sequences were aligned using the MAFFT v.7.110 online program under default parameters (Katoh and Standley 2013), and manually adjusted to allow maximum sequence similarity in Se-Al version.2.03a. (Rambaut 2000). Ambiguously aligned regions and gaps in alignment were excluded by Se-Al version.2.03a. (Rambaut 2000) before the phylogenetic analysis. Alignments were submitted to TreeBASE under accession number S25937. We conducted maximum likelihood (ML), most parsimonious (MP) and Bayesian inference (BI) analyses on the two datasets.

Maximum likelihood (ML) analysis of the dataset was carried out using RAxML 8.0.14 (Stamatakis 2014) and the GTRGAMMA substitution model with parameters unlinked. The ML bootstrap replicates (1000) were computed in RAxML using a rapid bootstrap analysis and search for the best-scoring ML tree. The ML trees were viewed with TreeView32 (Page 2001). Clades with bootstrap support (MLBS) ≥ 70% were considered as significant-supported (Hillis and Bull 1993).

A most parsimonious (MP) analysis was constructed with PAUP* 4.0b10. (Swofford 2002). The bootstrap values were generated using the following settings: 1000 replicate searches on all parsimoniously informative characters using 100 random sequence addition replications and TBR (tree-bisection reconnection) branch swapping algorithms in PAUP*. Tree statistics (TL), consistency index (CI), retention index (RI) and homoplasy index (HI) were also calculated. Tree was viewed with TreeView32 (Page 2001). Clades with bootstrap support (MPBS) ≥ 70% were considered to be significant (Hillis and Bull 1993).

Bayesian inference (BI) analyses was performed with MrBayes v3.1.2 (Ronquist and Huelsenbeck 2003) based on the best substitution models determined by MrModeltest 2.3 (Nylander 2004), which were GTR+I+G for the ITS dataset and SYM+I+G for the 28S dataset. Two independent runs of four chains were conducted for 4 000 000 for ITS and 2 000 000 for 28S datasets Markov Chain Monte Carlo generations using the default settings and sampled every 100 generations. The temperature value was lowered to 0.20, burn-in was set to 0.25, and the run was automatically stopped as soon as the average standard deviation of split frequencies reached below 0.01. A 50% majority-rule consensus tree was constructed and visualized with TreeView32 (Page 2001). Clades with Bayesian posterior probability (BPP) ≥ 0.95 were considered as significantly supported (Alfaro et al. 2003).

## Results

### Phylogenetic analysis

For ITS dataset, we comprehensively collected the ITS sequences of *Balsamia* and the fungi previously described as *Barssia*, and sequences that are high similarity to *Balsamia*. For 28S dataset, we collected all sequences of *Balsamia* and the fungi previously described as *Barssia*, and representative sequence of other genera of *Helvellaceae*. Sequences of each locus were aligned and analyzed separately.

**Figure 1.**
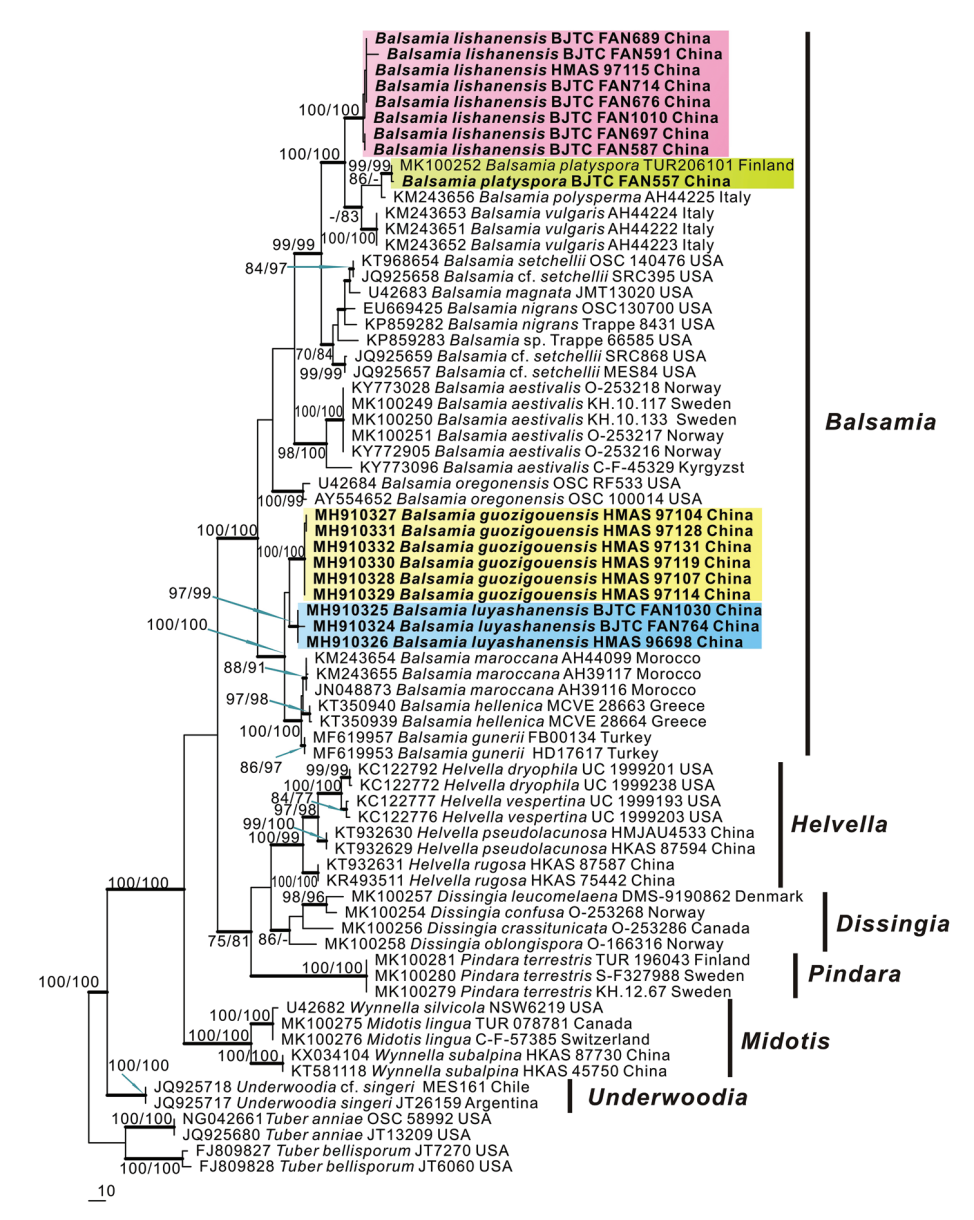
Phylogenetic tree generated from a maximum parsimonious analysis based on 28S sequences, showing the phylogenetic relationships of *Helvellaceae*. *Tuber anniae* and *T. bellisporum* are the outgroups. Maximum parsimonious bootstrap support values (≥ 70%) and maximum likelihood bootstrap support values (≥ 70%) are indicated above the nodes as MPBS/MLBS. Thick black branches received Bayesian posterior probabilities (BPP) ≥ 0.95. Novel sequences are printed in bold.

The 28S dataset contained 72 sequences (9 were newly gained in this study), and 4 from the outgroup *Tuber anniae* and *T. bellisporum*. The dataset had an aligned length of 886 characters, of which 578 were constant, 308 were variable, and 278 of these variable sites were informative. The maximum parsimony analysis resulted in one most parsimonious tree with a length (TL) of 842 steps, consistency index (CI) of 0.570, retention index (RI) of 0.896, homoplasy index (HI) of 0.430. MP, ML and BI analyses yielded similar tree topologies, and only the tree inferred from the MP analysis is shown (Fig. 1). The 28S sequences of *Balsamia* were grouped into a distinct clade with high supports (MPBS = 100%, MLBS = 100%, BPP = 1.00). The Chinese materials were well clustered in the *Balsamia* clade (Fig. 1), including the sequences of the fungi previously described as *Barrsia guozigouensis* L. Fan & Y.Y. Xu and *Barssia luyashanensis* L. Fan & Y.Y. Xu (Xu et al. 2018). Three distinct branches with strong supports can be recognized from Chinese collections, respectively representing *Balsamia guozigouensis*, *Balsamia luyashanensis*, and a new species *Balsamia lishanensis* proposed in this study. In addition, the Chinese sequence from BJTC FAN557 grouped together with a reliably identified sequence (MK100252) of *B. platyspora* (Hansen et al. 2019) with strong support value (MPBS = 99%, MLBS = 99%, BPP = 1.00), and they shared 99.83% 28S sequences similarity, indicating the Chinese specimen BJTC FAN557 was *B. platyspora*.

**Figure 2.**
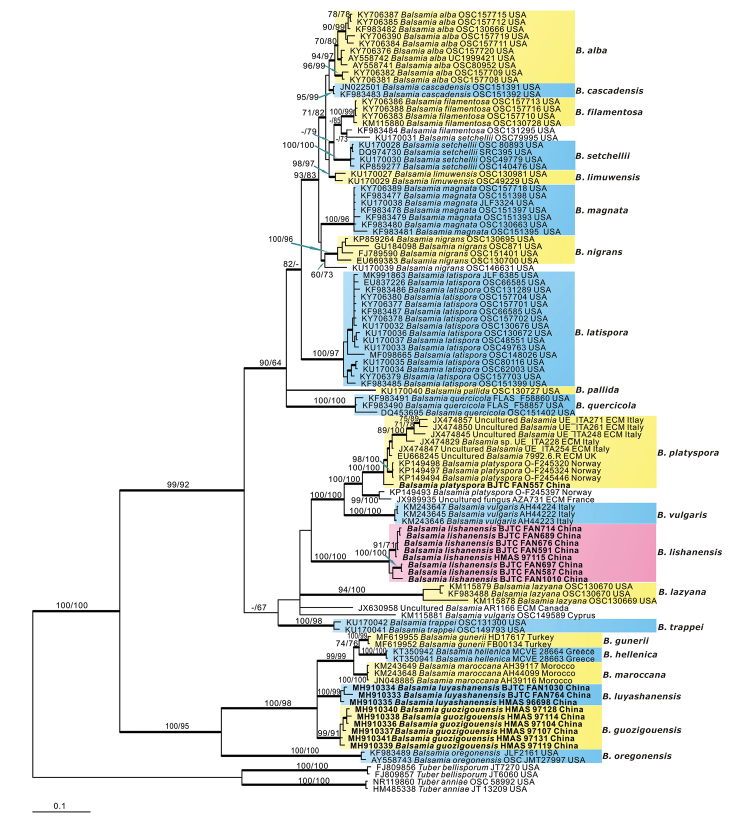
Phylogenetic tree generated from Bayesian analysis based on ITS sequences, showing the phylogenetic relationships of *Balsamia*. *Tuber anniae* and *T. bellisporum* are the outgroups. Maximum parsimonious bootstrap support values (≥ 70%) and maximum likelihood bootstrap support values (≥ 70%) are indicated above the nodes as MPBS/MLBS. Thick black branches received Bayesian posterior probabilities (BPP) ≥ 0.95. Novel sequences are printed in bold.

The ITS dataset contained 108 sequences (9 were newly gained in this study), and 4 from the outgroup *T. anniae* and *T. bellisporum*. The dataset had an aligned length of 1056 characters, of which 310 were constant, 745 were variable, and 622 of these variable sites were informative. The maximum parsimony analysis resulted in one most parsimonious tree with a length (TL) of 2220 steps, consistency index (CI) of 0.580, retention index (RI) of 0.900, homoplasy index (HI) of 0.420. MP, ML and BI analyses yielded similar tree topologies, and only the tree inferred from the Bayesian analysis is shown (Fig. 2). The ITS sequences of *Balsamia* were grouped into a distinct clade with high supports (MPBS = 100%, MLBS = 100%, BPP = 1.00), and the sequences from the Chinese collection unambiguously clustered in the *Balsamia* clade, including the sequences of the fungi previously described as *Barrsia guozigouensis* and *Barssia luyashanensis* (Xu et al. 2018) (Fig. 2). The sequences of all Chinese collections excepting specimen BJTC FAN557 were grouped into three independent clades with strong supports (Fig. 2), respectively representing *Balsamia guozigouensis*, *Balsamia luyashanensis* and a new species *Balsamia lishanensis* proposed in this study. The sequence of BJTC FAN557, which was identified as *B. platyspora* by morphology and 28S phylogeny (Fig. 1) in this study, formed a strong support clade together with nine European sequences isolated from ascomata of *Balsamia platyspora* or ectomycorrhizal root tips of *Balsamia*. These ten samples showed high sequences similarity so the clade was considered as representing *B. platyspora*.

Based on the above phylogenetic analyses (Figs 1, 2), we concluded that *Barrsia guozigouensis* and *Barssia luyashanensis* should be transferred to *Balsamia*. The clade of *B. lishanensis* was a distinct species and represented a new species. The specimen BJTC FAN557 should be recognized as the European *Balsamia platyspora*.

### Taxonomy

#### Balsamia lishanensis

Taxon classificationFungiPezizalesHelvellaceae

L. Fan & Y.Y. Xu sp. nov.

9008AC5E-59FF-5E20-985D-FFCF80F60306

834962

Figure 3

##### Etymology.

*lishanensis*, Lishan Mountain, referring to the locality where the type specimen was collected.

##### Holotype.

China. Shanxi Province, Yuanqu County, Lishan Mountain Shunwangping Scenic Area, alt. 2300 m, 17 October 2016, in soil under *Pinus armandii* Franch., M. Chen CM019 (BJTC FAN676).

Ascomata subglobose to irregularly subglobose, 3–14 × 2–12 mm in fresh, reddish brown when fresh, usually with some superficial furrows, surface covered with verrucose or fine warts, warts obtuse or pointed, 270–400 μm wide and 150–300 μm high. Odor light, mushroom flavor. Gleba solid, white to cream white, with numerous irregular canals and chambers of around 1 mm width. Peridium 150–350 μm thick, two-layered, outer layer pseudoparenchymatous, 90–190 μm thick, composed of 3–5 layers of reddish brown polygonal cells with 4-6 sides, cells 15–35 × 10–27 μm, walls 4.0–8.0 μm thick, the outermost cells reddish-brown, and gradually light-yellow to hyaline towards inner side; inner layer 60–150 μm thick, composed of interwoven hyphae, that is more or less parallel to the surface of peridium, hyphae hyaline, 2.5–6.0 μm wide. Paraphyses line the surface of chamber, arranged like a fence, 3–4 × 50 μm, but disorganized in the mature ascomata, usually not well-defined. Asci 8-spored, hyaline, citriform or fusiform, 55–80 × 27–38 μm (not including stalk), inamyloid, with a slender-stalk of 13.5–35 × 5–10 μm, spores irregularly arranged in ascus. Ascospores ellipsoid, smooth, hyaline, inamyloid, 20.6–25.6 × 12.9–15 μm (av. 23.5 × 14.0 μm), Q (L/I) = 1.55–1.80 (Qm = 1.68) (n = 30), usually containing one large oil drop and several small droplets.

**Figure 3.**
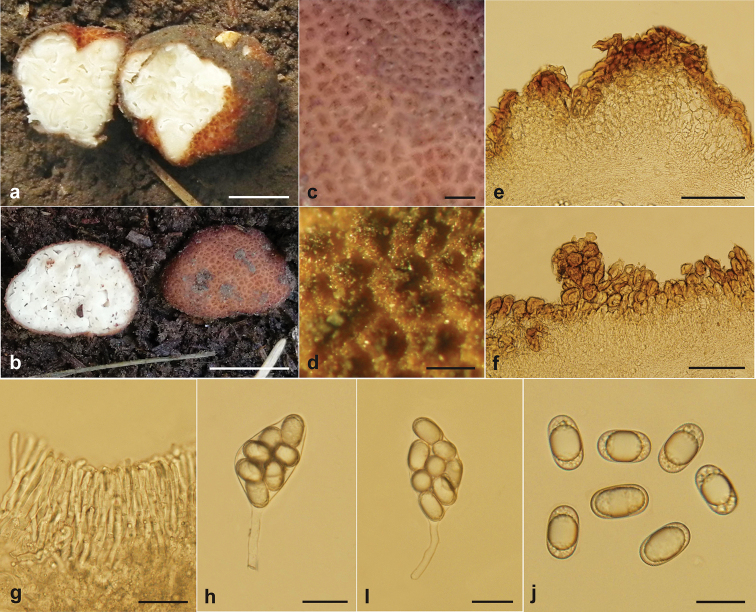
*Balsamia lishanensis* (BJTC FAN676, holotype) **a, b** ascomata **c** warts (when fresh) **d** warts (when dry) **e, f** peridium **g** paraphyses **h, i** mature ascus **j** ascospores. Scale bars: 5 mm (**a, b**); 500 μm (**c**); 300 μm (**d**); 100 μm (**e, f**); 25 μm (**g, h, i**); 20 μm (**j**).

##### Other materials examined.

China. Shanxi Province, Yuanqu County, Lishan Mountain Shunwangping Scenic Area, alt. 2300m, 16 August 2016, in soil under *Pinus armandii* Franch., K.B. Huang HKB003 (BJTC FAN587); *ibid.*, 16 August 2016, in soil under *Pinus armandii* Franch., B.D. He HBD014 (BJTC FAN 591); *ibid.*, 17 October 2016, in soil under *Pinus armandii* Franch., K.B. Huang HKB039 (BJTC FAN689); *ibid.*, 17 October 2016, in soil under *Pinus armandii* Franch., X.Y. Sang SXY015 (BJTC FAN697); *ibid.*, 17 October 2016, in soil under *Pinus armandii* Franch., K.B. Huang HKB031 (BJTC FAN714); China. Shanxi Province, Ningwu County, Xiaoshidong Village, Guancen Mountain, alt. 2000m, 12 October 2017, in soil under *Picea asperata* Mast., L.J. Guo GLJ001 (BJTC FAN1010); China. Gansu Province, Bailongjiang Forestry Bureau, Seventh Forest Farm, alt. 2500m, 14 July 2002, in soil under *Pinus* sp., D.J. Ren & M.S. Song 02-034 (HMAS 97115).

##### Notes.

*Balsamia lishanensis* was diagnosed by the combination of reddish brown ascomata covered with fine warts, the whitish gleba with numerous small chambers open to 1 mm, 3–5 layers peridium reddish brown polygonal cells and the smooth and regular ellipsoid ascospores with one large oil drop. There are four *Balsamia* species similar to *B. lishanensis* in morphology. Of them, *B. vulgaris* differed by its large ascospores of (23–) 26–32 (–36) × 11.5–14 (–16) μm, *Balsamia lazyana* and *B. trappei* by their narrow ascospores, which are 19.5–27 × 8–11.5 μm in *B. lazyana* and 24–26 × 11.5–13.5 μm in *B. trappei*, *B. platyspora* by its short-ellipsoid ascospores of 19–22–28 × 12–13–16 μm (ca. 20 × 13 μm). Phylogenetic analysis revealed that the sequences of *B. lishanensis* were grouped into an independent clade with strong support value (Figs 1, 2). DNA analysis showed that *B. lishanensis* shared less than 87.19% identity in ITS sequence with other *Balsamia* species. These supported the erection of the new species.

#### Balsamia platyspora

Taxon classificationFungiPezizalesHelvellaceae

Berk. Ann. Mag. Nat. Hist.13: 358(1884)

1FFB5716-A32E-5E5C-8277-62D7CFF29B7C

Figure 4

##### Materials examined.

China. Shanxi Province, Yuanqu County, Lishan Mountain Shunwangping Scenic Area, alt. 2200m, 16 August 2016, in soil under *Pinus armandii* Franch., Y.W. Wang WYW012 (BJTC FAN557).

##### Notes.

*Balsamia platyspora* is distributed in Europe, North America and Asia (Berkeley 1844; Gilkey 1939). In China, it is reported as early as 1990 from Shanxi Province based on morphological evidences (Liu and Tao 1990), but unfortunately, we have been unable to find the voucher specimen. In this study, our molecular analysis based on 28S sequences (Fig. 1) and ITS sequences (Fig. 2), and morphological studies confirmed the occurrence of this species in China based on the new collections from the Shanxi Province where this species was harvested originally by Liu and Tao (1990). *Balsamia platyspora* is mainly characterized by its minor subglobose ascomata, reddish brown to dark brown warts, white to yellowish white gleba with around 1 mm chambers, citriform or broadly elliptic asci, shortly elliptic ascospores 19–22–28 × 12–13–16 μm (ca. 20 × 13 μm) (Berkeley 1844; Hawker 1954; Pegler et al.1993), our specimen well matched the characteristics.

**Figure 4.**
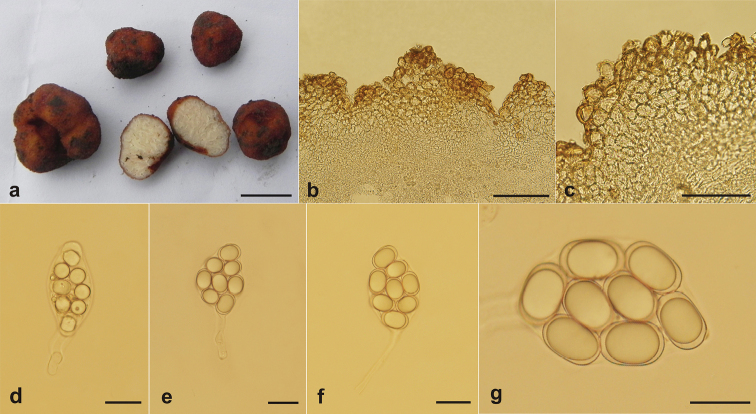
*Balsamia platyspora* (BJTC FAN557) **a** ascomata **b** peridium **c** warts **d** immature ascus **e, f** mature ascus **g** ascospores. Scale bars: 1 cm (**a**); 100 μm (**b, c**); 25 μm (**d, e, f**); 20 μm (**g**).

#### Balsamia guozigouensis

Taxon classificationFungiPezizalesHelvellaceae

(L. Fan & Y.Y. Xu) L. Fan & Y.Y. Xu. comb. nov.

61A0556F-0F88-56A3-9677-F0C3738112DB

834963

##### Basionym.

*Barssia guozigouensis* L. Fan & Y.Y. Xu, Phytotaxa 374(2): 135 (2018).

##### Holotype.

China. Xinjiang Autonomous Region, Huocheng County, Guozigou Forest Park, alt. 1800m, in soil under *Picea schrenkiana* Fisch. & C.A. Mey., 11 August 2003, W.P. Wu & M. S. Song 060 (HMAS 97107).

***Illustrations*** – Xu et al. (2018: Fig. 4)

##### Notes.

This species is recently described from Xinjiang Autonomous Region, China, under *Picea schrenkiana* Fisch. & C.A. Mey. So far it is known only from the type locality. *Balsamia guozigouensis* can be recognized by its distinctly warty ascomata, solid gleba with small and irregular chamber and irregularly clavate asci. Phylogenetically, it was closely related to *B. luyashanensis* (Fig. 2), but the latter differs in its ascomata with fine warts and gleba without chambers (Xu et al. 2018).

#### Balsamia luyashanensis

Taxon classificationFungiPezizalesHelvellaceae

(L. Fan & Y.Y. Xu) L. Fan & Y.Y. Xu. comb. nov.

5C88D99C-CE88-5B7B-8E5C-C97E66390EBD

834964

##### Basionym.

*Barssia luyashanensis* L. Fan & Y.Y. Xu, Phytotaxa 374(2): 134 (2018).

##### Holotype.

China. Shanxi Province, Ningwu County, Qiuqiangou Village, Luyashan Mountain, alt. 2100m, 25 August 2017, in soil under *Picea* sp., M. Chen CM023 (BJTC FAN764).

***Illustrations*** – Xu et al. (2018: fig. 3)

##### Notes.

*Balsamia luyashanensis* is also recently described from the Luyashan Mountain of Shanxi Province, China, under *Picea* sp. So far it is known only from the type locality. The species can be recognized by its red brown ascomata with fine warts, gleba without chambers and irregularly clavate asci (Xu et al. 2018). The species was similar in appearance of ascomata to *B. gunerii* and *B. hellenica* but *B. gunerii* can be separated by its subglobose to ovoid ascospores and gleba with irregularly sinuous, labyrinth-like veins (Doğan et al. 2018; Hansen et al. 2019), while *B. hellenica* by its ovoid ascospores (Kaounas et al. 2015; Hansen et al. 2019).

### Key to Chinese species of *Balsamia*

**Table:** 

1	Ascomata with an obvious apical depression	**2**
–	Ascomata without an obvious apical depression	**3**
2	Surface with distinct warts, solid gleba scattered with some small and irregular chambers	***B. guozigouensis***
–	Surface with fine warts, solid gleba without chambers	***B. luyashanensis***
3	Ascospores long-ellipsoid, (20.6–25.6 × 12.9–15 μm, Q = 1.55–1.80)	***B. lishanensis***
–	Ascospores short-ellipsoid, 19–22–28 × 12–13–16 μm (ca. 20 × 13 μm) (Hawker 1954)	***B. platyspora***

## Supplementary Material

XML Treatment for Balsamia lishanensis

XML Treatment for Balsamia platyspora

XML Treatment for Balsamia guozigouensis

XML Treatment for Balsamia luyashanensis

## Supplementary materials

Supplementary material 1 Table S1. Taxa used in this study and their GenBank accession numbers for ITS and 28S sequence data Data type Microsoft Word Document (.docx) https://binary.pensoft.net/file/417834This dataset is made available under the Open Database License (http://opendatacommons.org/licenses/odbl/1.0/). The Open Database License (ODbL) is a license agreement intended to allow users to freely share, modify, and use this Dataset while maintaining this same freedom for others, provided that the original source and author(s) are credited.Yu-Yan Xu, Xiang-Yuan Yan, Ting Li, Li Fan
