# Supplementary material for: A taxonomic reassessment of the genus Balsamia from China
Source: MycoKeys. 2020 Jun 4;67:81–94. doi: 10.3897/mycokeys.67.50068 (PMC7289895; doi:10.3897/mycokeys.67.50068)
Supplement: Supplementary material 1 — Table S1. Taxa used in this study and their GenBank accession numbers for ITS and 28S sequence data [file mycokeys-67-081-s001.docx]

Table 1. Taxa used in this study and their GenBank accession numbers for ITS and 28S sequence data.

| **Fungal taxon** | **Specimen voucher** | **Locality** | **ITS** | **28S** | **References** |
| --- | --- | --- | --- | --- | --- |
| *Balsamia aestivalis* | O-253218 | Norway | — | KY773028 | Skrede et al. 2017 |
|  | KH.10.117 | Sweden | — | MK100249 | Hansen et al. 2019 |
|  | KH.10.133 | Sweden | — | MK100250 | Hansen et al. 2019 |
|  | O-253217 | Norway | — | MK100251 | Hansen et al. 2019 |
|  | O-253216 | Norway | — | KY772905 | Skrede et al. 2017 |
|  | C-F-45329 | Kyrgyzstan | — | KY773096 | Skrede et al. 2017 |
| *B*. *alba* | OSC 157715 | USA | KY706387 | — | Southworth et al. 2018 |
|  | OSC 157712 | USA | KY706385 | — | Southworth et al. 2018 |
|  | OSC 157719 | USA | KY706390 | — | Southworth et al. 2018 |
|  | OSC 157711 | USA | KY706384 | — | Southworth et al. 2018 |
|  | OSC 130666 | USA | KF983482 | — | Southworth et al. 2018 |
|  | OSC 157720 | USA | KY706376 | — | Southworth et al. 2018 |
|  | OSC 157709 | USA | KY706382 | — | Southworth et al. 2018 |
|  | OSC 157708 | USA | KY706381 | — | Southworth et al. 2018 |
|  | OSC 80952 | USA | AY558741 | — | Southworth et al. 2018 |
|  | UC 1999421 | USA | AY558742 | — | Southworth et al. 2018 |
| *B*. *cascadensis* | OSC 151392 | USA | KF983483 | — | Southworth et al. 2018 |
|  | OSC 151391 | USA | JN022501 | — | Southworth et al. 2018 |
| *B*. *filamentosa* | OSC 157716 | USA | KY706388 | — | Southworth et al. 2018 |
|  | OSC 157713 | USA | KY706386 | — | Southworth et al. 2018 |
|  | OSC 157710 | USA | KY706383 | — | Southworth et al. 2018 |
|  | OSC 130728 | USA | KM115880 | — | Southworth et al. 2018 |
|  | OSC 131295 | USA | KF983484 | — | Southworth et al. 2018 |
| *B*. *gunerii* | FB 00134 | Turkey | MF619952 | MF619957 | Doğan et al. 2018 |
|  | HD 17617 | Turkey | MF619955 | MF619953 | Doğan et al. 2018 |
| *B*. *guozigouensis* | HMAS 97104 | China | MH910336 | MH910327 | Xu et al. 2018 |
|  | HMAS 97107 | China | MH910337 | MH910328 | Xu et al. 2018 |
|  | HMAS 97114 | China | MH910338 | MH910329 | Xu et al. 2018 |
|  | HMAS 97119 | China | MH910339 | MH910330 | Xu et al. 2018 |
|  | HMAS 97128 | China | MH910340 | MH910331 | Xu et al. 2018 |
|  | HMAS 97131 | China | MH910341 | MH910332 | Xu et al. 2018 |
| *B*. *hellenica* | MCVE 28663 | Greece | KT350941 | KT350940 | Kaounas et al. 2015 |
|  | MCVE 28664 | Greece | KT350942 | KT350939 | Kaounas et al. 2015 |
| *B*. *latispora* | OSC 148026 | USA | MF098665 | — | Southworth et al. 2018 |
|  | OSC 157702 | USA | KY706378 | — | Southworth et al. 2018 |
|  | OSC 157704 | USA | KY706380 | — | Southworth et al. 2018 |
|  | OSC 66585 | USA | KF983487 | — | Southworth et al. 2018 |
|  | OSC 157701 | USA | KY706377 | — | Southworth et al. 2018 |
|  | OSC 49763 | USA | KU170033 | — | Southworth et al. 2018 |
|  | OSC 48551 | USA | KU170037 | — | Southworth et al. 2018 |
|  | OSC 130672 | USA | KU170036 | — | Southworth et al. 2018 |
|  | OSC 131289 | USA | KF983486 | — | Southworth et al. 2018 |
|  | OSC 130676 | USA | KU170032 | — | Southworth et al. 2018 |
|  | OSC 151399 | USA | KF983485 | — | Southworth et al. 2018 |
|  | OSC 157703 | USA | KY706379 | — | Southworth et al. 2018 |
|  | OSC 62003 | USA | KU170034 | — | Southworth et al. 2018 |
|  | OSC 80116 | USA | KU170035 | — | Southworth et al. 2018 |
|  | OSC 66585 | USA | EU837226 | — | Southworth et al. 2018 |
|  | JLF 6358 | USA | MK991863 | — | Unpublished |
| *B*. *lazyana* | OSC 130670 | USA | KM115879 | — | Southworth et al. 2018 |
|  | OSC 130670 | USA | KF983488 | — | Southworth et al. 2018 |
|  | OSC 130669 | USA | KM115878 | — | Southworth et al. 2018 |
| *B*. *limuwensis* | OSC 49229 | USA | KU170029 | — | Southworth et al. 2018 |
|  | OSC 130981 | USA | KU170027 | — | Southworth et al. 2018 |
| ***B*. *lishanensis*** | **BJTC FAN587** | **China** | **MT232721** | **MT232903** | **This study** |
|  | **BJTC FAN591** | **China** | **MT232899** | **MT232911** | **This study** |
|  | **BJTC FAN676** | **China** | **MT232907** | **MT232902** | **This study** |
|  | **BJTC FAN689** | **China** | **MT232905** | **MT232914** | **This study** |
|  | **BJTC FAN697** | **China** | **MT232908** | **MT232912** | **This study** |
|  | **BJTC FAN714** | **China** | **MT232901** | **MT232913** | **This study** |
|  | **BJTC FAN1010** | **China** | **MT232900** | **MT232910** | **This study** |
|  | **HMAS 97115** | **China** | **MT232904** | **MT232909** | **This study** |
| *B*. *luyashanensis* | HMAS 96698 | China | MH910335 | MH910326 | Xu et al. 2018 |
|  | BJTC FAN764 | China | MH910333 | MH910324 | Xu et al. 2018 |
|  | BJTC FAN1030 | China | MH910334 | MH910325 | Xu et al. 2018 |
| *B*. *magnata* | OSC 157718 | USA | KY706389 | — | Southworth et al. 2018 |
|  | OSC 151393 | USA | KF983479 | — | Southworth et al. 2018 |
|  | OSC 151398 | USA | KF983477 | — | Southworth et al. 2018 |
|  | JLF 3324 | USA | KU170038 | — | Southworth et al. 2018 |
|  | OSC 130663 | USA | KF983480 | — | Southworth et al. 2018 |
|  | OSC 151397 | USA | KF983478 | — | Southworth et al. 2018 |
|  | OSC 151395 | USA | KF983481 | — | Southworth et al. 2018 |
|  | JMT 13020 | USA | — | U42683 | O’Donnell et al. 1997 |
| *B*. *maroccana* | AH 39116 | Morocco | JN048885 | JN048873 | Alvarado et al. 2011 |
|  | AH 39117 | Morocco | KM243649 | KM243655 | Crous et al. 2014 |
|  | AH 44099 | Morocco | KM243648 | KM243654 | Crous et al. 2014 |
| *B*. *nigrans* | OSC 151401 | USA | FJ789590 | — | Southworth et al. 2018 |
|  | OSC 130695 | USA | KP859264 | KP859282 | Southworth et al. 2018 |
|  | SOC 871 | USA | GU184098 | — | Southworth et al. 2018 |
|  | OSC 130700 | USA | EU669383 | EU669425 | Southworth et al. 2018 |
|  | OSC 146631 | USA | KU170039 | — | Unpublished |
| *B*. *oregonensis* | JLF2161 | USA | KF983489 | — | Southworth et al. 2018 |
|  | OSC JM27997 | USA | AY558743 | — | Izzo et al. 2005 |
|  | OSC 100014 | USA | — | AY544652 | Unpublished |
|  | OSC RF533 | USA | — | U42684 | O’Donnell et al. 1997 |
| *B*. *pallida* | OSC 130727 | USA | KU170040 | — | Southworth et al. 2018 |
| *B*. *platyspora* | O-F245320 | Norway | KP149498 | — | Unpublished |
|  | O-F245446 | Norway | KP149494 | — | Unpublished |
|  | O-F245324 | Norway | KP149497 | — | Unpublished |
|  | TUR 206101 | Finland | — | MK100252 | Hansen et al. 2019 |
|  | **BJTC FAN557** | **China** | **MT232906** | **MT229143** | **This study** |
|  | O-F245397 | Norway | KP149493 | — | Southworth et al. 2018 |
| *B*. *polysperma* | AH 44225 | Italy | — | KM243656 | Crous et al. 2014 |
| *B*. *quercicola* | FLAS_F58860 | USA | KF983491 | — | Southworth et al. 2018 |
|  | FLAS_F58857 | USA | KF983490 | — | Southworth et al. 2018 |
|  | OSC 151402 | USA | DQ453695 | — | Southworth et al. 2018 |
| *B*. *setchellii* | SRC 395 | USA | DQ974730 | — | Southworth et al. 2018 |
|  | OSC 80893 | USA | KU170028 | — | Southworth et al. 2018 |
|  | OSC 140476 | USA | KP859277 | KT968654 | Southworth et al. 2018 |
|  | OSC 49779 | USA | KU170030 | — | Southworth et al. 2018 |
|  | OSC 79995 | USA | KU170031 | — | Southworth et al. 2018 |
| *B*. cf. *setchellii* | SRC868 | USA | — | JQ925659 | Bonito et al. 2013 |
| *B*. cf. *setchellii* | SRC395 | USA | — | JQ925658 | Bonito et al. 2013 |
| *B*. cf. *setchellii* | MES84 | USA | — | JQ925657 | Bonito et al. 2013 |
| *B*. *trappei* | OSC 131300 | USA | KU170042 | — | Southworth et al. 2018 |
|  | OSC 149793 | USA | KU170041 | — | Southworth et al. 2018 |
| *B*. *vulgaris* | AH44222 | Italy | KM243645 | KM243651 | Crous et al. 2014 |
|  | AH44224 | Italy | KM243647 | KM243653 | Crous et al. 2014 |
|  | AH44223 | Italy | KM243646 | KM243652 | Crous et al. 2014 |
|  | OSC 149589 | Cyprus | KM115881 | — | Southworth et al. 2018 |
| *Balsamia* sp. | UE_ITA228 | Italy | JX474829 | — | Benucci et al. 2014 |
| *Balsamia* sp. | Trappe 66585 | USA | — | KP859283 | Unpublished |
| *Uncultured Balsamia* | UE_ITA271 | Italy | JX474857 | — | Benucci et al. 2014 |
| *Uncultured Balsamia* | UE_ITA261 | Italy | JX474850 | — | Benucci et al. 2014 |
| *Uncultured Balsamia* | UE_ITA248 | Italy | JX474845 | — | Benucci et al. 2014 |
| *Uncultured Balsamia* | UE_ITA254 | Italy | JX474847 | — | Benucci et al. 2014 |
| *Uncultured Balsamia* | 7992.6.R | UK | EU668245 | — | Bidartondo et al. 2008 |
| *Uncultured Balsamia* | AR1166 | Canada | JX630958 | — | Timling et al. 2012 |
| Uncultured fungus | AZA731 | France | JX989935 | — | Roy et al. 2013 |
| *Dissingia confusa* | O-253268 | Norway | — | MK100254 | Hansen et al. 2019 |
| *D*. *crassitunicata* | O-253286 | Canada | — | MK100256 | Hansen et al. 2019 |
| *D*. *leucomelaena* | DMS-9190862 | Denmark | — | MK100257 | Hansen et al. 2019 |
| *D*. *oblongispora* | O-166316 | Norway | — | MK100258 | Hansen et al. 2019 |
| *Helvella dryophila* | UC 1999201 | USA | — | KC122792 | Nguyen et al. 2013 |
|  | UC 1999238 | USA | — | KC122772 | Nguyen et al. 2013 |
| *H*. *pseudolacunosa* | HKAS 87594 | China | — | KT932629 | Ariyawansa et al. 2015 |
|  | HMJAU 4533 | China | — | KT932630 | Ariyawansa et al. 2015 |
| *H. rugosa* | HKAS 75442 | China | — | KR493511 | Ariyawansa et al. 2015 |
|  | HKAS 87587 | China | — | KT932631 | Ariyawansa et al. 2015 |
| *H*. *vespertina* | UC 1999193 | USA | — | KC122777 | Nguyen et al. 2013 |
|  | UC 1999203 | USA | — | KC122776 | Nguyen et al. 2013 |
| *Midotis lingua* | TUR 078781 | Canada | — | MK100275 | Hansen et al. 2019 |
|  | C-F-57385 | Switzerland | — | MK100276 | Hansen et al. 2019 |
| *Pindara terrestris* | KH.12.67 | Sweden | — | MK100279 | Hansen et al. 2019 |
|  | S-F327988 | Sweden | — | MK100280 | Hansen et al. 2019 |
|  | TUR 196043 | Finland | — | MK100281 | Hansen et al. 2019 |
| *Underwoodia singer* | JT 26159 | Argentina | — | JQ925717 | Bonito et al. 2013 |
| *U*. cf. *singeri* | MES 161 | Chile | — | JQ925718 | Bonito et al. 2013 |
| *Wynnella silvicola* | NSW 6219 | USA | — | U42682 | O’Donnell et al. 1997 |
| *W. subalpine* | HKAS 45750 | China | — | KT581118 | Zhao et al. 2016 |
|  | HKAS 87730 | China | — | KX034104 | Zhao et al. 2016 |
| *Tuber anniae* | JT 13209 | USA | HM485338 | JQ925680 | Bonito et al. 2013 |
|  | OSC 58992 | USA | NR119860 | NG042661 | Bonito et al. 2013 |
| *T*. *bellisporum* | JT 6060 | USA | FJ809857 | FJ809828 | Bonito et al. 2010 |
|  | JT 7270 | USA | FJ809856 | FJ809827 | Bonito et al. 2010 |

Note: “—” shows no sequence in the GenBank database. GenBank accession numbers for sequences gained in this study are in boldface.
